# Identification and partial characterization of a novel serpin from *Eudiplozoon nipponicum* (Monogenea, Polyopisthocotylea)

**DOI:** 10.1051/parasite/2018062

**Published:** 2018-12-05

**Authors:** Pavel Roudnický, Jiří Vorel, Jana Ilgová, Michal Benovics, Adam Norek, Lucie Jedličková, Libor Mikeš, David Potěšil, Zbyněk Zdráhal, Jan Dvořák, Milan Gelnar, Martin Kašný

**Affiliations:** 1 Department of Botany and Zoology, Faculty of Science, Masaryk University Kamenice 753/5 62500 Brno Czech Republic; 2 Veterinary Research Institute Hudcova 296/70 62100 Brno Czech Republic; 3 Department of Parasitology, Faculty of Science, Charles University Viničná 7 12844 Prague 2 Czech Republic; 4 Central European Institute of Technology, Masaryk University Kamenice 753/5 62500 Brno Czech Republic; 5 National Centre for Biomolecular Research, Faculty of Science, Masaryk University Kamenice 753/5 62500 Brno Czech Republic; 6 School of Biological Sciences, Medical Biology Centre, Queen’s University Belfast 97 Lisburn Road Belfast BT9 7BL United Kingdom; 7 Department of Zoology and Fisheries, Faculty of Agrobiology, Food and Natural Resources, Czech University of Life Sciences in Prague Kamýcká 129 16521 Prague Czech Republic

**Keywords:** *Eudiplozoon nipponicum*, fish parasite, monogenea, Platyhelminths, serpin, inhibitor

## Abstract

*Background*: Serpins are a superfamily of serine peptidase inhibitors that participate in the regulation of many physiological and cell peptidase-mediated processes in all organisms (e.g. in blood clotting, complement activation, fibrinolysis, inflammation, and programmed cell death). It was postulated that in the blood-feeding members of the monogenean family Diplozoidae, serpins could play an important role in the prevention of thrombus formation, activation of complement, inflammation in the host, and/or in the endogenous regulation of protein degradation.

*Results*: *In silico* analysis showed that the DNA and primary protein structures of serpin from *Eudiplozoon nipponicum* (EnSerp1) are similar to other members of the serpin superfamily. The inhibitory potential of EnSerp1 on four physiologically-relevant serine peptidases (trypsin, factor Xa, kallikrein, and plasmin) was demonstrated and its presence in the worm’s excretory-secretory products (ESPs) was confirmed.

*Conclusion*: EnSerp1 influences the activity of peptidases that play a role in blood coagulation, fibrinolysis, and complement activation. This inhibitory potential, together with the serpin’s presence in ESPs, suggests that it is likely involved in host-parasite interactions and could be one of the molecules involved in the control of feeding and prevention of inflammatory responses.

## Abstract (fr)

Contexte : Les serpines sont une super-famille d’inhibiteurs de sérine peptidases qui participent, dans tous les organismes, à la régulation de nombreux processus physiologiques et à médiation par les peptidases cellulaires (par exemple la coagulation sanguine, l’activation du complément, la fibrinolyse, l’inflammation et la mort cellulaire programmée). Il a été postulé que chez les Monogènes de la famille Diplozoidae, qui sont hématophages, les serpines pourraient jouer un rôle important dans la prévention de la formation de thrombus, l’activation du complément, l’inflammation chez l’hôte et/ou la régulation endogène de la dégradation des protéines. Résultats : Une analyse *in silico* a montré que l’ADN et les structures primaires protéiques de la serpine d’*Eudiplozoon nipponicum* (EnSerp1) sont similaires aux autres membres de la superfamille des serpines. Le potentiel inhibiteur d’EnSerp1 sur quatre sérine peptidases physiologiquement pertinentes (la trypsine, le facteur Xa, la kallikréine et la plasmine) a été démontré et sa présence dans les produits excréteurs de sécrétion du ver (ESP) a été confirmée. Conclusion : EnSerp1 influence l’activité des peptidases qui jouent un rôle dans la coagulation sanguine, la fibrinolyse et l’activation du complément. Ce potentiel inhibiteur, ainsi que la présence de la serpine dans les ESP, suggèrent qu’elle est probablement impliquée dans les interactions hôte-parasite et pourrait être l’une des molécules impliquées dans le contrôle de l’alimentation et la prévention des réponses inflammatoires.

## Introduction

Almost 40% of peptidases deposited in the MEROPS database (Release 12.0) are serine peptidases (SPs) and up to 70% of all deposited inhibitors are SP inhibitors, 25% of which are serpins (*Ser*ine *P*eptidase *In*hibitors) [13, 21, 84]. Although nucleotide/amino acid sequences of serpins are not highly conserved across organisms (AA similarity around 30% [13]), their secondary structures typically consist of three *β*-sheets (A, B, C) and eight to nine *α*-helices [21, 29]. Serpins are usually 350–450 AAs long, which corresponds to 40–60 kDa [20, 61, 88]. Importantly, a characteristic sequence motif, a reactive centre loop (RCL) containing a scissile bond between P1 and P1’ residues, is responsible for the serpins’ inhibitory effect towards peptidases. This structure is bound and cleaved by the active site of target SP, which results in a distortion and inactivation of the enzyme [21, 65].

In addition to having an inhibitory effect on SPs [14, 21, 32, 34, 61], some serpins are also capable of inhibiting peptidases of other catalytic types, such as cysteine peptidases: a human SCCA1 serpin interacts with cathepsins K, L, and S [87], while cowpox virus CrmA serpin inhibits interleukin-1*β* converting enzyme, which is a caspase [51]. Some serpins lack inhibitory properties and have other biological roles. They function for instance as hormone transporters, which is the case of the human corticosteroid-binding globulin [24], as chaperones, in particular chaperone HSP47 (from bacteria to mammals) [39], or as putative storage proteins, which is the case of chicken ovalbumin [21].

Among parasitic flatworms, there are many reports of both SPs and serpin inhibitors. These enzymes have various functions including immune evasion, collagenolytic activity, and digestion of the host’s tissue, which is related to host invasion and migration through the body [2, 12, 16, 17, 52, 70, 78, 79]. It has also been reported that serpins probably play a key role in immune evasion or immunomodulation in, e.g., *Echinococcus multilocularis* [64], *Schistosoma mansoni* [22], and *Schistosoma japonicum* [98]; another serpin from *Schistosoma haematobium* inhibits blood coagulation during feeding [9], in *Schistosoma mansoni* another one may acts as a regulator of cercarial elastase activity [60], while a serpin from *Clonorchis sinensis* participates in the development of the specific life stages of the parasite [43].

Within the Monogenea, data about SPs and their inhibitors are scarce. So far, only a single report exists on potential SP activity in whole worm extracts from *Neobenedenia girellae* [28]; the authors hypothesise that SPs could play an important role in the life cycle of the parasite, influencing processes such as egg laying by adults and the swimming of oncomiracidia. Our experimental organism, a diplozoid sanguinivorous monogenean *Eudiplozoon nipponicum* Goto, 1891 (Polyopisthocotylea) is a common ectoparasite that inhabits the gills of cyprinid fish, most frequently the common carp (*Cyprinus carpio*). In monogeneans, blood digestion takes place only partly in the gut lumen [40] and for the most part, digestion occurs in specialised hematin cells of the gastrodermis (intestinal epithelium) [53]. Little is known about the molecules secreted by monogeneans during blood uptake. This contrasts with knowledge on some haematophagous ectoparasites belonging to other taxa, such as ticks, which produce two functionally predominant groups of molecules in their salivary glands – antihemostatic and immunomodulatory factors. In these organisms, serpins have been identified as effective regulators of host inflammatory processes, complement activation, blood clotting, and platelet aggregation [15, 57, 80, 81]. The present work is part of our on-going research to identify and understand the key peptidases and their inhibitors produced by *E. nipponicum* [37, 40, 41]. In the present study, we report the first evidence of a serpin molecule produced by Monogenea and present a description of its basic molecular and functional characteristics.

## Methods

### Parasite material

Adults of *E. nipponicum* were collected from freshly sacrificed specimens of *Cyprinus carpio* caught in the Mušov lowland reservoir, Czech Republic (48°53′12″N, 16°34′37″E). Isolation of the individual worms from the gills was performed under a TH4-200 stereomicroscope (Olympus). Living worms were taxonomically identified under a BX50 light microscope (Olympus) equipped with a differential interference contrast (Nomarski DIC), and species identity was confirmed by sequencing [62].

For the preparation of excretory-secretory products (ESPs), worms (app. 100 individuals) were gently washed in 10 mM PBS pH 7.2 and incubated in fresh buffer of the same composition for 3 h at room temperature (RT). Samples were concentrated on Amicon^®^ Ultra 3K centrifugal filters (Merck Millipore). Crude worm extract (CWE) was prepared by gently washing *E. nipponicum* adults (five individuals) in 10 mM PBS pH 7.2 and manually homogenised in 0.1 M acetate buffer pH 5, sonicated with BioLogics 150 VT ultrasonic homogeniser (60% amplitude, three cycles of 30 s), centrifuged (16 000×*g*, 20 min, 4 °C), and the supernatant was collected.

Protein concentration in ESP and CWE was determined using a Quaint-iT^TM^ Protein Assay Kit (Life Technologies) and SpectraMax i3 fluorometer (Molecular Devices). Prior to their use, samples were stored at −80 °C.

### *In silico* sequence analyses

A nucleotide sequence with a complete open reading frame (ORF) encoding EnSerp1 was obtained from assembled transcripts from *E. nipponicum* transcriptome (the *E. nipponicum* Transcriptome Shotgun Assembly project has been deposited at DDBJ/EMBL/GenBank under Accession Number GFYM00000000) [7]. Additionally, two other serpin-coding sequences were identified; EnSerp1 (E_nip_trans_58759_m.371742) was selected for this study due to the higher number of transcript copies (983 150.64 transcripts per million), whereas the number of copies of other serpin genes was one (E_nip_trans_50452_m.341543) and two (E_nip_trans_65948_m.402754) orders lower. Deduced protein sequence was analysed using the basic local alignment search tool (BLAST), available at the National Center for Biotechnology Information website (http://ncbi.nlm.nih.gov). From all serpins used for the phylogenetic analysis (see below), four sequences with the highest level of similarity to EnSerp1 were chosen to analyse the conserved areas in the alignment using Geneious 7.1.9 software (Biomatters Ltd.). Other analyses of serpin sequences were performed with Proteomics and sequence analysis tools (http://ca.expasy.org/tools). Secondary and tertiary structure predictions were carried out via web portal Phyre2, set to intensive mode [47]. Subsequent graphic visualisation and analysis of the EnSerp1 molecule were performed with the UCSF Chimera software tool, version 1.10.2 [77] in combination with Modeller 9.16 [86].

### Phylogenetic analysis

For the phylogenetic analysis, amino acid sequences of serpin homologs from 13 platyhelminth taxa were aligned with EnSerp1 using ClustalW software [92]. The list of sequences obtained from GenBank, including the EnSerp1 sequence, is presented in Supplementary file 1. The predicted AA sequence of a serpin from *Gyrodactylus salaris* (scf7180006950201) was obtained from genome data deposited in the WormBase ParaSite database [30, 31] (accession no. PRJNA244375). Sequence alignment was checked and optimised manually to avoid ambiguously aligned regions. ModelGenerator v. 0.851 [46] was employed to test 12 evolutionary models and to approximate the most suitable one using the Bayesian information criterion (BIC). Phylogenetic trees were inferred according to Bayesian inference (BI) and Maximum likelihood (ML) in the MrBayes 3.2 [85] and RaxML v 8.1.X programmes, respectively [89]. Bayesian inference was carried out using the Metropolis-coupled Markov chain Monte Carlo (MC^3^) algorithm with two parallel runs, using one cold and three hot chains. The computation ran for 10^6^ generations and sampling tree topologies were constructed every 10^2^ generations. Thirty percent of all saved trees were discarded as a relative burn-in period based on a standard deviation split frequency value (<0.01). Posterior probabilities were calculated as the frequency of samples recovering any particular clade. Clade support for ML was assessed by 1000 bootstrap pseudoreplicates, subtree pruning and regrafting (SPR), and nearest neighbour interchange (NNI) branch swapping algorithms were applied.

### RNA, cDNA, and plasmid preparation

*E. nipponicum* RNA was isolated using a High Pure RNA Tissue Kit (Roche). A Transcriptor First Strand cDNA Synthesis Kit (Roche) with anchored-oligo(dT)_18_ primer was used for reverse transcription of RNA to cDNA.

PCR was performed as described previously [41], using specific primers for the *E. nipponicum* EnSerp1 gene (EnSerp1 forward: 5′-ATGTGCGCTTGTCCTAATAG-3′, EnSerp1 reverse: 5′-TTATTTGGAAGGTTCTGGATCC-3′) and higher annealing temperature (55 °C). The PCR products (1200 bp) were resolved in 1% agarose gel, purified by a MinElute PCR Purification Kit (Qiagen) and sub-cloned into a pJET1.2 cloning vector (CloneJET PCR Cloning Kit, Thermo Scientific), which was used to transform *E. coli* XL-1 Blue (Novagen). pJET1.2 constructs were isolated using a High Pure Plasmid Isolation Kit (Roche) and sequenced with pJET1.2 forward and reverse primers from the kit (DNA Sequencing Laboratory, Faculty of Science, Charles University, Prague).

Expression vector pET-22b(+) containing the EnSerp1 insert (1200 bp) was synthesised by GeneScript company, with the coding sequence for His-tag on C-terminus. *E. coli* TOP10 cells were transformed by this construct for DNA plasmid propagation (heat shock 42 °C for 45 s). Plasmids were isolated using a High Pure Plasmid Isolation Kit (Roche) and used to transform ArcticExpress (DE3) Competent Cells (Agilent Technologies), according to the manufacturer’s instructions. Positive colonies were verified by sequencing and sequences analysed by Geneious 7.1.9.

### Expression of recombinant *E. nipponicum* serpin (rEnSerp1) and its purification

Overnight culture (5 mL, OD ≈ 2) was inoculated into 500 mL of a fresh LB medium (L3022, Sigma-Aldrich) with ampicillin (100 μg/mL). Bacteria were grown at a temperature of 30 °C at 200 rpm until OD = 0.8 was reached, then cooled down to 12 °C and induced by IPTG (0.5 mM) for 18 h. Harvested cells were lysed in a binding buffer (specifications below) by sonication on ice, and subsequently centrifuged at 10 000 × *g* for 40 min at 4 °C. Soluble rEnSerp1 was purified from the supernatant by immobilised metal affinity chromatography (IMAC) using Ni^2+^ HisTrap columns (1 mL, GE Healthcare) connected to FPLC (ÄKTA, GE Healthcare).

Buffers used in the individual stages of purification were composed as follows: binding buffer – 50 mM Tris-HCl pH 8, 10 mM imidazole, 300 mM NaCl, 5% glycerol, 0.05% Tween; elution buffer – 50 mM Tris-HCl pH 8, 500 mM imidazole, 300 mM NaCl, 5% glycerol, 0.05% Tween. Protein purity was evaluated in a 4–15% TGX SDS-PAGE gel under reducing conditions (Bio-Rad Mini-Protean). In a sample buffer, wells contained 5 μg of protein. Gel documentation was performed in a calibrated densitometer GS-900™ (Bio-Rad). Recombinant EnSerp1 was deprived of imidazole and salts using PD-10 columns (GE Healthcare), and then transferred into a 100 mM HEPES buffer (pH 7.5 with 300 mM NaCl, 10 mM CaCl_2_, and 0.05% Brij L23), i.e. the same buffer that was used for inhibition assays. Protein concentration was determined using a Quaint-iT^TM^ Protein Assay Kit (Life Technologies) and a SpectraMax i3 fluorometer (Molecular Devices).

### LC-MS analyses

A major band of a size corresponding to rEnSerp1 (around 45 kDa) was manually excised from the 1D gel, destained, and incubated with trypsin (Promega) at 37 °C for 2 h. Peptides were extracted from the gel using a solution of 50% acetonitrile with 5% formic acid prior to LC-MS/MS analysis.

E/S products of *E. nipponicum* were also analysed using LC-MS/MS. A sample of ESP was processed using a filter-assisted sample preparation technique (FASP; using Ultracel-10 kDa Membrane units), and the resulting peptide mixture was analysed using the LC-MS/MS system.

LC-MS/MS analyses of all peptide mixtures were carried out on an RSLCnano liquid chromatograph connected online to an Orbitrap–Elite mass spectrometer (Thermo Scientific). In-gel or E/S product digests were analysed using a 40 or 100 min long nonlinear gradient, respectively (mobile phase A: 0.1% formic acid in water; mobile phase B: 0.1% formic acid in 80% acetonitrile). MS and MS/MS (HCD fragmentation) spectra were recorded in an Orbitrap analyser (resolution 60 000 at 400 m/z and 15 000 at 400 m/z, respectively).

MS/MS data were processed using Proteome Discoverer software, version 1.4, and database searches executed using the Mascot search engine (version 2.6, Matrix Science). MS/MS data for in-gel digest were searched against a cRAP contaminant database (based on http://www.thegpm.org/crap/), local database containing a serpin sequence and UniRef100 database (all taxonomies, no restrictions, ftp://ftp.ebi.ac.uk/pub/databases/uniprot/uniref/uniref100/uniref100.fasta.gz; version 2017-06, 112 447 146 sequences in total). The cRAP database and concatenated database of *E. nipponicum* transcripts, *C. carpio* proteins (downloaded from NCBI), and the UniRef100 protein database for platyhelminths (downloaded from UniProt.org) were used to acquire E/S sample data. Modifications for all database searches were set as follows: oxidation (M), deamidation (N, Q), and acetylation (Protein N-term) as optional modifications, with carbamidomethylation (C) as a fixed modification. Enzyme specificity was tryptic or semitryptic for in-gel digest and E/S sample data, respectively, with one allowed miscleavage. Only peptides with a Mascot Ion Score above 40, rank 1, and at least six amino acids long were considered during the confirmation of presence of rEnSerp1 and/or EnSerp1 proteins.

### Production of antibodies

Anti-rEnSerp1 antibodies were produced in two ICR/CD1 mice (ENVIGO) injected intraperitoneally with 50 μg of rEnSerp1 cut as a band from a polyacrylamide gel after SDS-PAGE. The strip of gel containing the antigen was washed and homogenised, as described previously [40]. The mice were boosted twice at 14-day intervals by intramuscular injections of purified antigen (15 μg) in sterile saline. Blood was collected from the animals under a deep ketamine/xylazine anaesthesia 14 days after the last injection, and sera were obtained by centrifugation. Control (pre-immune) sera were collected from the same mice prior to immunisation.

### Western blot analysis

The presence of EnSerp1 in ESP and CWE, and specificity of anti-rEnSerp1 antibodies were tested on Western blots. ESP, CWE, and rEnSerp1 samples were run on SDS-PAGE under the conditions described above. Proteins were transferred onto a PVDF membrane (Bio-Rad) using Trans-Blot Turbo™ (Bio-Rad) with the protocol set to a constant 1.3 A for 10 min. After overnight blocking in 10 mM PBS, pH 7.2, containing 5% non-fat milk, 2.5% BSA, and 0.05% Tween 20, the membranes were incubated with mouse anti-rEnSerp1 antisera (1:50) in a blocking buffer for 1.5 h. Mouse pre-immune sera were used as a negative control. After washing in PBS (3 × 5 min), the membranes were incubated with peroxidase-conjugated goat anti-mouse IgG (1:400, AP124P, Sigma-Aldrich) in the blocking buffer for 1 h. After the final washing (3 × 5 min), reactions were visualised using an Opti-4CN™ substrate kit (Bio-Rad), according to the manufacturer’s instructions. Signal intensity was read in a calibrated densitometer GS-900™ (Bio-Rad).

### Peptidase inhibition assays

The inhibitory effect of rEnSerp1 was tested with commercially available peptidases (Sigma-Aldrich) in the presence of the respective fluorogenic peptide substrates in black, flat-bottom microtiter plates (Nunc): human thrombin (T6884) + VPR-AMC (B9385, Sigma-Aldrich), bovine factor X activated (F9302) + IEGR-AMC (B9936, Sigma-Aldrich), human plasmin (10602361001) + ALK-AMC (A8171, Sigma-Aldrich), human plasma kallikrein (K2638) + FR-AMC (I-1160.0050, Bachem), porcine elastase (E7885) + AAPV-AMC (S4760, Sigma-Aldrich), and porcine trypsin (T6567) + GPR-AMC (I-1150.0025, Bachem). Mixtures of the buffer (300 mM NaCl, 10 mM CaCl_2_, 0.05% Brij L23, pH 7.5) and substrate served as blanks. Substrate and enzyme concentrations were 20 nM and 10 nM, respectively. Each peptidase was preincubated with 0.425 μM EnSerp1 for 10 min at RT before adding a corresponding substrate. The relative fluorescence of released aminomethyl coumarin was measured in kinetic cycles at 30 s intervals for 45 min in the SpectraMax i3 fluorometer (Molecular Devices) at excitation and emission wavelengths 355 and 460 nm, respectively. Measurements were carried out in triplicate and the experiment was repeated twice. These data were normalised between zero and 100% of the peptidase activity. Resulting graphs represent an arithmetic average of all measurements. Standard deviation is expressed as an error bar.

Trypsin and factor Xa were also assayed in reaction with a bacterial extract without the presence of rEnSerp1 to eliminate the possibility of a false result due to possible bacterial contaminants. The sample of a bacterial extract (ArcticExpress *E*. *coli* without an inserted gene coding the rEnSerp1) was prepared in the same way as the rEnSerp1, i.e. by adopting IMAC and FPLC during the sample preparation.

## Results

### *In silico* analysis of EnSerp1 sequence

**Figure 1.**
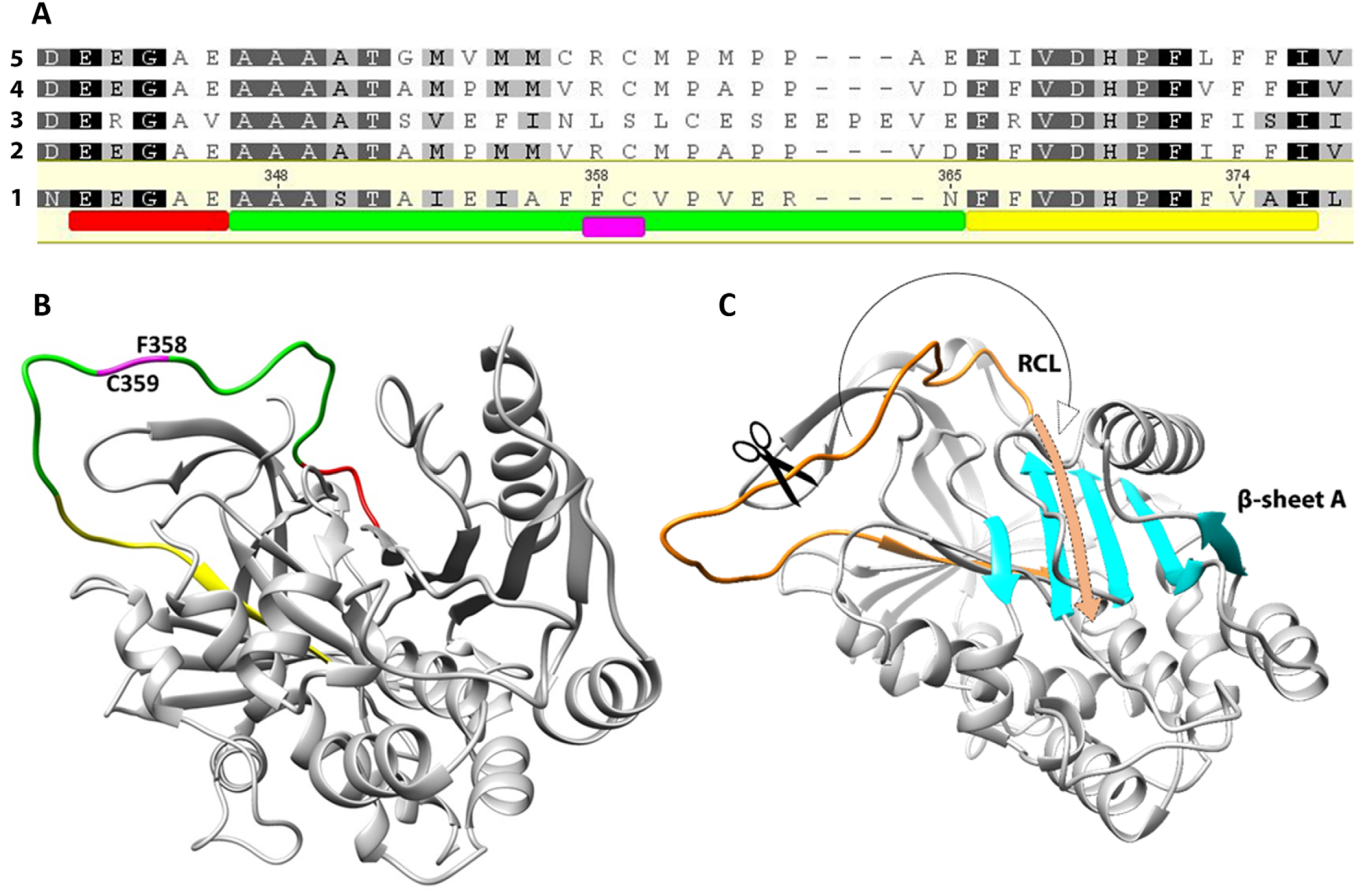
(A) Alignment of the EnSerp1 sequence with four of the most similar serpin sequences of other platyhelminths: 1, EnSerp1 from *Eudiplozoon nipponicum* (GenBank: MF288891.1); 2, *Echinococcus multilocularis* (GenBank: CDS35969.1); 3, *Schistosoma haematobium* (GenBank: XP_012797533.1); 4, *Echinococcus granulosus* (GenBank: CDS22753.1); 5, *Taenia solium* (GenBank: ATG83400.1). Conserved motifs characteristic for serpins are highlighted. The serpin motif (E^342^ – E^346^) shown in red is part of the reactive centre loop (RCL, A^347^ – N^365^), shown in green. Immediately after RCL, follows serpin signature (F^366^ – I^376^) in yellow. Scissile bond is situated within the RCL between P1 (F^358^) and P1’ (C^359^) residue, shown in magenta. (B) Predicted 3D structure of EnSerp1. Coloured areas of the molecule correspond to the sequence highlighted in Figure 1A. (C) RCL and *β*-sheet A. After the peptidase cleaves the scissile bond within RCL (in orange), the residual part of RCL is incorporated as a new strand into *β*-sheet A (in cyan).

The complete ORF of the EnSerp1 gene submitted to the database (GenBank: MF288891.1) was determined to be a 1200 bp long sequence encoding 399 amino acids. The predicted molecular weight of the protein was 44.65 kDa (45.64 with His-tag) and the calculated pI was 5.59. No signal peptide was found. A conserved motif typical of serpins was identified through alignment with four of the most closely similar serpin sequences from other platyhelminths: *Echinococcus multilocularis* (GenBank: CDS35969.1), *Schistosoma haematobium* (GenBank: XP_012797533.1), *Echinococcus granulosus* (GenBank: CDS22753.1), and *Taenia solium* (GenBank: ATG83400.1]). The most relevant parts of the sequence are highlighted in Figure 1A. Two additional serpin sequences identified in *E. nipponicum* transcriptome (E_nip_trans_50452_m.341543 and E_nip_trans_65948_m.402754) shared 97.3% and 94.5% similarity with EnSerp1, respectively.

Six templates for predicting EnSerp1’s 3D structure based on heuristics to maximise confidence, percentage identity, and amino acid alignment coverage were selected using the Phyre2 web tool [47] (Fig. 1B). For templates, we used the following molecules: in two cases antithrombin III (PDB: 1ATT and 2B5T) [42, 67], in another two cases plasminogen activator inhibitor-1 (PAI-1) (PDB: 1LJ5 [unpublished Stein PE and Baek K, 2002] and 4DTE [3]), in one case heparin cofactor II (PDB: 1JMO) [4], and likewise in one case the molecule known as chaperone Hsp47/Serpinh1 (PDB: 3ZHA) [95]. According to these templates, the most probable 3D structure for EnSerp1 was built with an over 90% confidence for 95% of residues. The theoretical tertiary structure is composed of nine right-hand-coiled *α*-helices and three antiparallel *β*-sheets.

### Phylogenetic analysis

**Figure 2.**
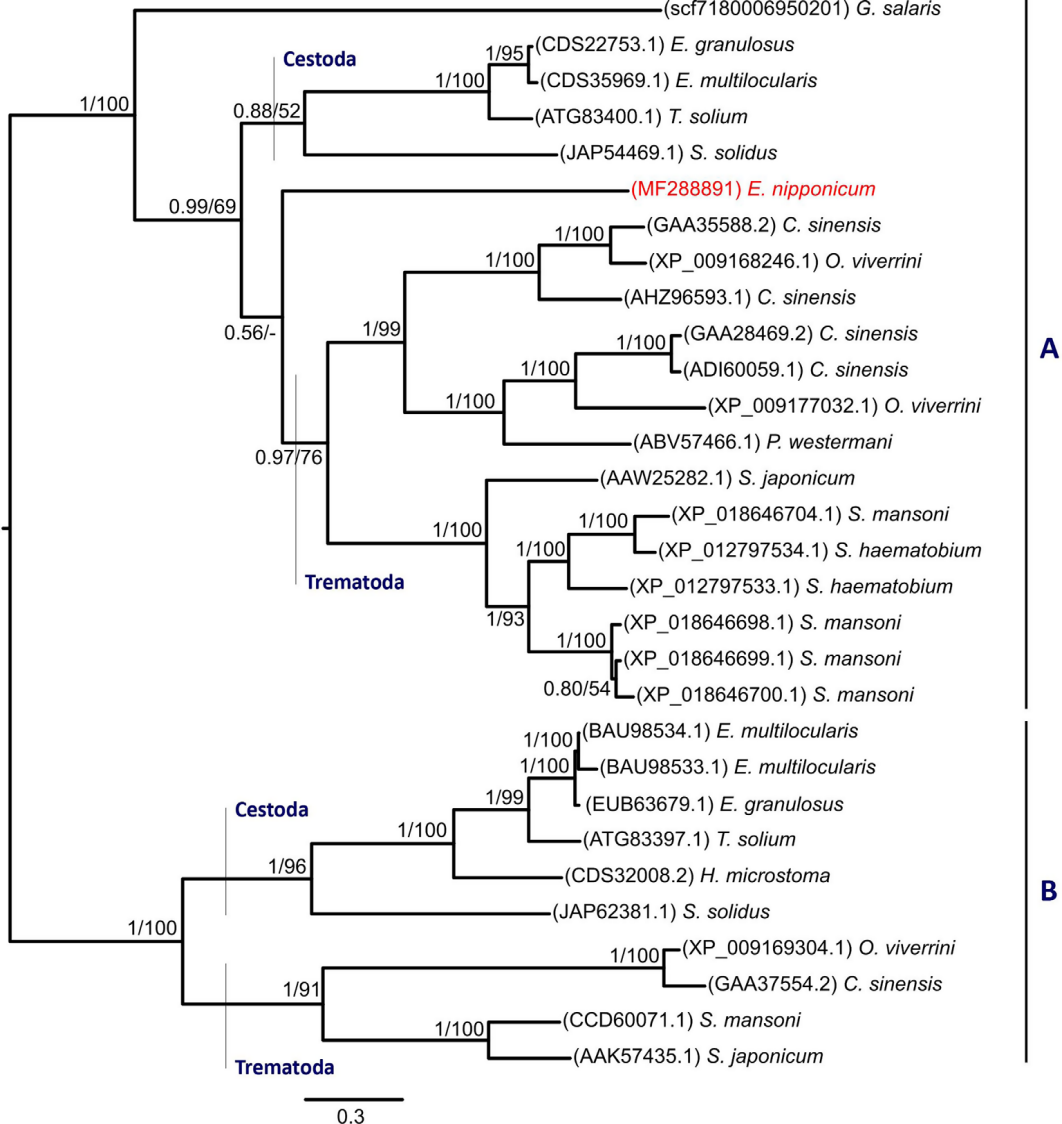
A phylogram of platyhelminth serpin homologs, Bayesian inference analysis. Values along the branches indicate posterior probabilities and bootstrap values resulting from Bayesian inference and Maximum likelihood analyses, respectively. Proportional lengths of the branches correspond to the expected number of amino acid substitutions per site. The resulting tree is mid-point rooted in order to visualise the clustering of representative subfamilies. Newly obtained *Eudiplozoon nipponicum* serpin homolog (EnSerp1) is labelled red.

The resulting 429-residue-long amino acid alignment built from 30 sequences contained 25 invariable sites. An LG+I+G [56] model was calculated by ModelGenerator as the optimal evolutionary model and applied in Bayesian inference and Maximum likelihood analyses. Both methods yielded trees with identical topologies. The resulting BI tree with posterior probabilities (PP) obtained by BI analysis and Maximum likelihood bootstrap values (BS) along the branches is presented in Figure 2.

The resulting cladogram divided platyhelminth serpin homologs in two well-supported groups (subfamilies) (PP = 1, BS = 100), A and B (see Fig. 2). EnSerp1 clustered with platyhelminth serpin homologs in Group A. Its position as a sister clade to serpins of the Trematoda class within the clade is weakly supported by BI and not supported by ML analyses. Serpin homologs of trematodes form a well-supported monophyletic clade within both subfamilies. Within Group A, serpin orthologs of cestodes form a well-supported clade, with the exception of a serpin ortholog from *Schistocephalus solidus*, whose position is supported only moderately or weakly (PP = 0.88, BS = 52). Cestode serpin homologs of Group B, on the other hand, in both analyses form a well-supported clade that includes all representatives.

### The expression of recombinant serpin and MS analysis

**Figure 3.**
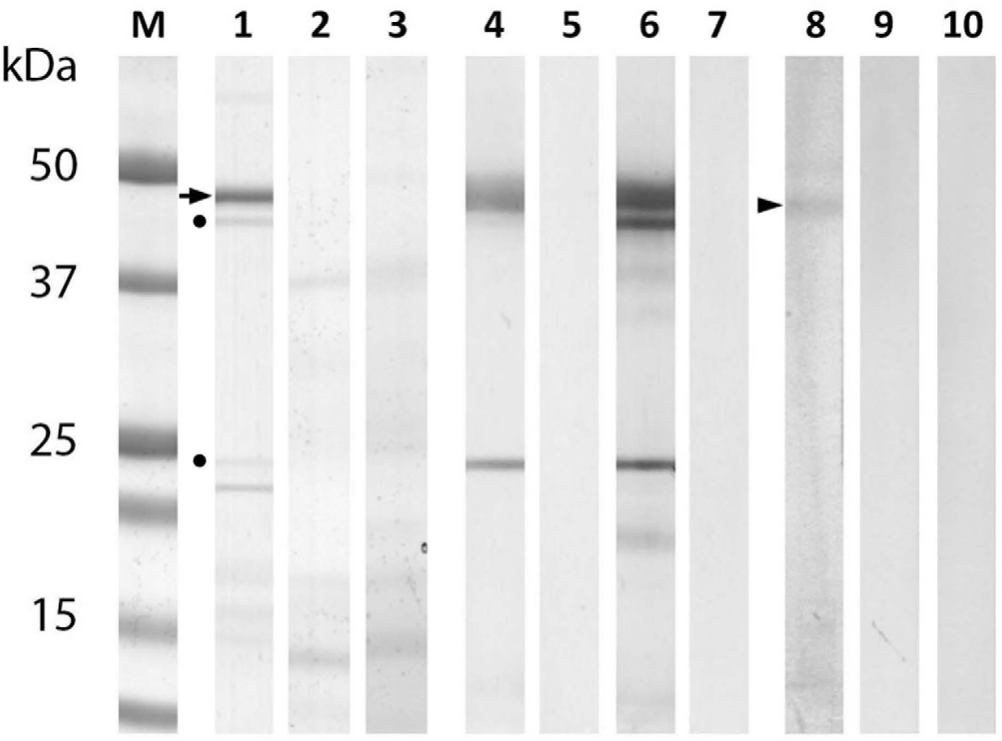
SDS PAGE and Western blots with rEnSerp1, ESP and CWE. Lines 1–3, 1D gel. Lines 4–10, Western blot. M, protein standard; 1, rEnSerp1; 2, ESP; 3, CWE sample; 4, rEnSerp1 with anti-HIS antibodies; 5, rEnSerp1 without primary antibodies; 6, rEnSerp1 with anti-rEnSerp1 sera; 7, rEnSerp1 with pre-immune sera; 8, ESP with anti-rEnSerp1 sera; 9, ESP with pre-immune sera; 10, CWE with anti-rEnSerp1 sera. Arrow points to the expected recombinant EnSerp1 band. Arrowhead points to the natural form of EnSerp1 in the ESP sample. Dots indicate fragmented parts of rEnSerp1.

A 45 kDa band corresponding to the theoretical size of rEnSerp1 appeared in the gel after electrophoresis (Fig. 3). Mass spectrometry data analysis had shown that EnSerp1 is the best-scoring protein in the given band (27 peptides and sequence coverage 75%). A search against the UniRef100 database did not indicate the presence of any other major protein component in this band. An MS examination of other bands from the gel showed that two minor bands represented fragments of rEnSerp1, while the remaining bands were bacterial contaminants.

E/S products were also subjected to an LC-MS analysis to verify the presence of EnSerp1. It was identified in this sample by 20 peptides that covered 63% of the amino acid sequence (data not shown). The identity of 45 kDa rEnSerp1 on blots was confirmed with mouse anti-rEnSerp1 antibodies and commercial mouse anti-His-tag antibodies (Invitrogen, cat. no. 372900). Anti-rEnSerp1 antibodies also reacted with the band of a corresponding size in ESP, but not in CWE (Fig. 3).

### Peptidase inhibition assays

**Figure 4.**
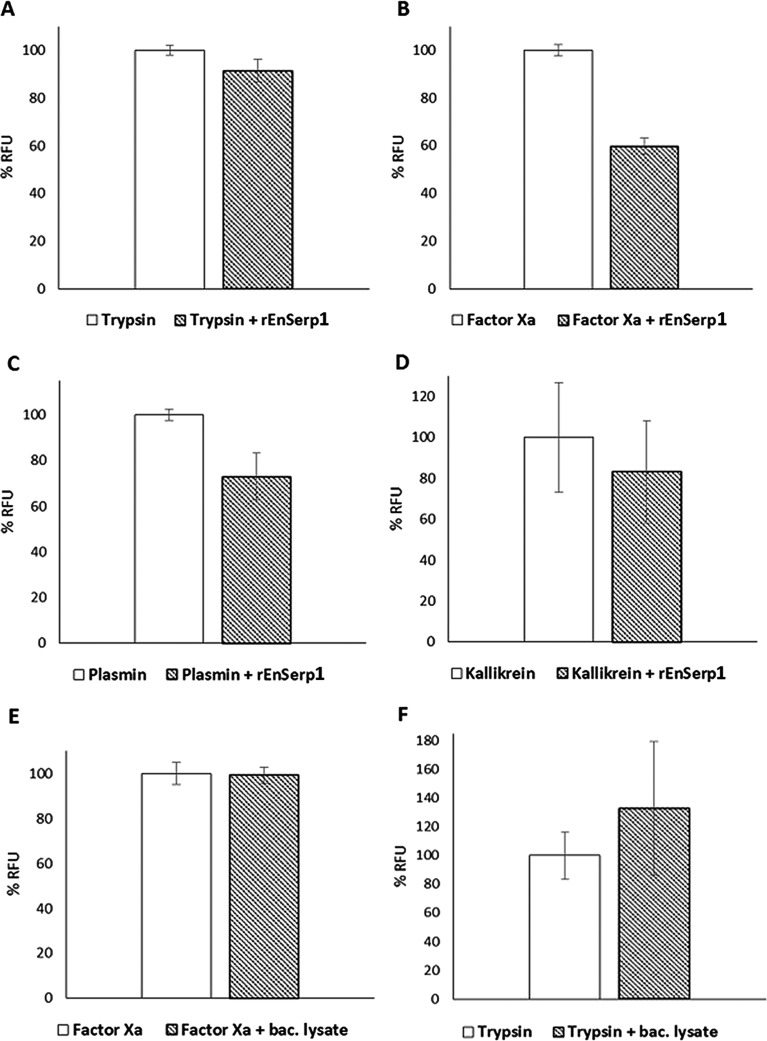
The inhibitory effect of rEnSerp1 on selected SPs. (A) trypsin; (B) factor Xa; (C) plasmin; (D) plasma kallikrein. Results of assays with a bacterial lysate instead of rEnSerp1: (E) factor Xa and F, trypsin. Data are expressed as a mean value ± standard deviation.

The inhibitory effect of rEnSerp1 on four peptidases (trypsin, factor Xa, plasmin, and plasma kallikrein) was determined by a kinetic measurement of residual peptidase activities in the presence of the appropriate fluorogenic substrates. rEnSerp1 inhibited trypsin activity by 8.5 ± 3.8%, factor Xa by 40.2 ± 2.5%, plasmin by 27.1 ± 7.7%, and plasma kallikrein by 17 ± 19.2% (Figs. 4A–4D). It had no effect on thrombin and pancreatic elastase (not shown). Measurement with an rEnSerp1-free bacterial lysate showed either no inhibition or even enhancement of substrate cleavage due to possible residual activity of a low amount of bacterial peptidases (Figs. 4E and 4F).

## Discussion

The *Eudiplozoon nipponicum* (family Diplozoidae), a haematophagous ectoparasite of the common carp, belongs to the highly radiated class of Monogenea. So far, this invasive parasite species was investigated mainly in studies focused on taxonomy, phylogeny, zoogeography, morphology/anatomy, basic biology, ecology, ecotoxicology, and pathogenicity [54, 93, 100]. The *E. nipponicum* can have a pathogenic effect on fish. In particular, blood intake by worms attached to the gills can eventually lead to hypochromic microcytic anaemia [45], while the small ruptures of gill lamellae can become a gateway for secondary bacterial and viral infections [5, 71, 97]. While participation of parasite molecules in pathological processes has been described for other helminths [27, 44], our knowledge of the particulars of parasite-host interactions at a molecular level and the functional contexts for particular monogenean molecules is lagging far behind. The only study so far that focused on monogenean SPs is a general study by Hirazawa et al. [28], while other studies investigated the primary structure of cathepsin L of the mucophagous *Neobenedenia melleni* [82] and proposed functional characterisation of cysteine and aspartic peptidases of *E*. *nipponicum*, which seem to play a role in blood digestion and vitellogenesis [41]. Two other studies related to *E. nipponicum* molecules were published recently; the first dealt with stefin, an inhibitor of cysteine peptidases, which can inhibit the activity of cathepsins L3 and B, thus possibly regulating their functions in the digestive process and reproduction, respectively [37]. The second study focused on a broad range of cysteine peptidases, their annotation and functional characterisation, while also proposing extracellular digestion in blood-feeding monogeneans [40].

The present study is the first characterisation of monogenean serpin, an inhibitor of SPs. In a transcriptome of adult *E. nipponicum*, we identified three highly similar serpin-coding sequences, possibly paralogs resulting from gene duplications. The most abundant one, EnSerp1, was selected for heterologous expression in *E*. *coli*. The recombinant form of *E. nipponicum* serpin (rEnSerp1) possesses 399 amino acids (without His-tag). This corresponds to the usual size of serpins in other organisms (350–400 AA) [20, 61, 88]. The molecular structure of the EnSerp1 protein molecule includes conserved regions that are specific to the serpin family: a serpin motif (E^342^–E^346^), serpin signature (F^366^–I^376^), and a hypervariable reactive centre loop (RCL) located in-between them (see also Figs. 1A and 1B). Alongside the serpin motif, EnSerp1 also contains a three-alanine region (A^347^–A^349^) assumed to be of crucial importance in serpins with inhibitory function because it has the ability to change conformation after cleavage of the scissile bond [38]. Differences in this region are usually linked to other general properties of serpins, such as hormone binding [76], chaperoning [69], or protein storage functions [38]. Based on 3D modelling, we predicted that EnSerp1’s secondary and tertiary structures are also characteristic of serpins: they fold into an N-terminal mostly helical domain and a C-terminal *β*-barrel-like domain consisting of nine *α*-helices and three *β*-sheets in total. Another crucial structure clearly recognisable in the model is the RCL connected to a *β*-sheet A. This is important for proper inhibitory function, because the RCL is incorporated into the *β*-sheet A during conformational changes, after cleavage of the scissile bond (Fig. 1C) [21, 29, 59]. These properties are present in the earlier described serpin-like structures, such as antitrypsin (PDB: 7API) [18], ovalbumin (PDB: 1OVA) [90], antichymotrypsin (PDB: 2ACH) [6], and plasminogen activator 1(PAI1) (PDB: 1C5G) [66], as well as in the more recently described ones, such as serpin18 (PDB: 4R9I) [23], SRPN18 (PDB: 5C98) [63], and vaspin (PDB: 4IF8) [26].

Phylogenetic analyses have shown that EnSerp1 is related to other platyhelminth serpins. In early pre-analyses (including investigations of mammalian, fish, insect, and reptilian sequences), we observed a clustering of platyhelminth serpins into one clade (data not shown). Within this clade, serpins seemed to form two main serpin groups, A and B (see Fig. 2). The crucial distinction between these two groups is determined by the most significant insertion/deletion between amino acid residues 100–112 (for the alignment of all serpins, see Supplementary file 1). Sequences that form Group B have an insertion in this region, which suggests a possible differentiation of their purpose during evolution, although unfortunately, we have not been able to find any studies dealing with the specific functions associated with this region. According to the phylogenetic tree, EnSerp1 forms a separate branch in Group A (that lacks the abovementioned sequence region) and serpins belonging to this group are more closely related to trematode serpins than to their orthologs from the class Cestoda. This is an interesting observation, because monogeneans are generally believed to be evolutionarily closer to cestodes than to trematodes [58, 73]. According to our phylogenetic tree, another relevant sequence of the monogenean species *G. salaris* is not related to either of the two classes (i.e. neither Cestoda nor Trematoda) and forms a separate branch within Group A. This surprising outcome could be influenced by the fact that the *G. salaris* serpin sequence is not completely accurate since it was only predicted from the genome without any further reconfirmation.

Among all the serpins used for the phylogenetic analysis, only three had been characterised in more detail (GenBank: AAK57435.1 [98]; ADI60059.1 [99]; ABV57466.1 [35]), and only in one (GenBank: ABV57466.1) it had been shown that it has the ability to inhibit SPs [35]. This serpin was obtained from the lung trematode *Paragonimus westermani* and its sequence, like that of EnSerp1, belongs to Group A.

Serpins typically form complexes at 1:1 molar ratio with target peptidase while using a unique irreversible mechanism of inhibition: they act as a suicide substrate [13]. This process is initiated by the formation of a covalent intermediate serpin-peptidase complex, which is then stabilised by a conformational translocation of the bound peptidase to the opposite pole of the serpin, where it distorts the peptidase active site [55, 88]. This translocation of peptidase is related to the incorporation of the RCL into the abovementioned β-sheet A. In our kinetic measurements, we tested the inhibitory effect of rEnSerp1 on several SPs, namely trypsin, factor Xa, plasmin, kallikrein, elastase, and thrombin. The last two were not inhibited at all. Incomplete inhibition of peptidases by rEnSerp1 in our experiments could be in part due to the origin of the peptidases we tested. Our measurements were conducted with commercial peptidases derived from porcine pancreas (trypsin and elastase), bovine plasma (factor Xa), and human plasma (thrombin, plasmin, and kallikrein), whereas the most probable targets of EnSerp1 are host peptidases from the common carp. Using a BLAST, we discovered only a 47% match between amino acid sequences of bovine factor Xa (GenBank: NP_001073682.1) and its predicted homolog from the carp genome (GenBank: KTF93720.1). Although the sequence similarity overall is relatively low, the crucial inhibitory potential is often related to the structure of the binding site within the reactive centre of a particular inhibitor molecule [25]. A similar situation was also observed for the other peptidases: human thrombin (GenBank: AAH51332.1) showed a 53% match with carp (GenBank: KTF81317.1), human plasmin (GenBank: AAA36451.1) a 57% match with carp (GenBank: KTF80813.1), porcine elastase (GenBank: 1207237A) a 62% match with carp (GenBank: KTG34206.1), and porcine trypsin (GenBank: NP_001156363.1) a 65% match with carp trypsin (GenBank: BAL04385.1). For plasma kallikrein, we could not find any homologous peptidase because in teleosts, the plasma kallikrein-kinin system cascade is absent [96].

Incomplete inhibition of selected SPs could also be associated with other factors. In addition to the relative specificity of rEnSerp1 discussed above, one can also assume that not all rEnSerp1 molecules in the examined sample were properly folded in an active conformation. It has been reported that due to an unstable native state, serpins are prone to polymerisation [49]. Polymerisation and incorrect folding can occur even at a physiological pH and temperature [75]. It is also possible that the inhibitory effect of serpins could be increased by including a cofactor into the reaction; for example, the inhibitory capacity of human antithrombin against thrombin was optimal when the reaction included the glycosaminoglycan heparin [8, 33, 74, 83]. Taking this into consideration, we tried to inhibit factor Xa by rEnSerp1 in the presence of heparin (heparin sodium salt, 20 nM, Zentiva), but no inhibitory effect was observed (data not shown). In order to detect any possible residual contamination with inhibitory effect in the sample of purified rEnSerp1 expressed in bacteria, a control with bacterial lysate instead of rEnSerp1 was tested. In this case, enhanced hydrolytic activity towards the substrate was observed, which might reflect the presence of active bacterial peptidases in the sample that would cleave the GPR [68]. Nevertheless, the bacterial lysate sample alone did not show any inhibitory effect, which indicates that the inhibition achieved in our measurements was related solely to rEnSerp1.

Activated factor X is an important SP of the blood coagulation cascade: it converts prothrombin to thrombin, which then cleaves fibrinogen to fibrin that forms a blood clot. Among haematophagous parasites, inhibitors that affect this SP tend to play a significant role in feeding [11, 19, 36, 48, 50]. Plasmin participates in fibrinolysis by cleaving fibrin clots and is involved in host defence mechanisms by regulating immune cells such as monocytes, macrophages, and dendritic cells [91]. Plasma kallikrein, too, is involved in fibrinolysis: it generates plasmin from plasminogen. In mammals, its other important function is a conversion of kininogens to kinins, polypeptides which regulate inflammation and blood pressure [10]. As noted above, however, plasma kallikrein and high-molecular-weight kininogen are not present in the teleosts [96], which makes any speculations about a possible interaction between monogenean serpins and the kallikrein/kinin system of the host moot. Nevertheless, the observed influence of rEnSerp1 on factor Xa and plasmin has one other possible interpretation, namely that it plays a role in inhibiting the host complement cascade. It has been shown that both of these SPs can convert C3 and C5 components to their active forms (C3a, C5a) [1, 72], thereby propagating inflammation. Given that in fish, innate immunity is of great importance and inflammation is the first response [94], EnSerp1’s interference with the peptidolytic activity of factor Xa and plasmin may be a way of preventing the host immune system from damaging the parasite’s tissue.

## Conclusion

In comparison to the other parasitic classes of platyhelminths, biochemical research tended to pay relatively little attention to Monogenea, although they include some species that have an economic impact, e.g. the *Gyrodactylus salaris*, the salmon fluke, which represents a significant problem for salmon farming. This situation seems to be slowly changing now with several recently published papers focused on proteins that function at the interface between the monogenean blood-feeder *E. nipponicum* and its fish host [37, 40, 41]. The results of the present study contribute to a growing body of knowledge regarding these molecules and processes in which they participate. Our characterisation of the molecular/biochemical properties of a serpin from *E. nipponicum* (EnSerp1) is the first record of such a group of peptidase inhibitors in Monogenea. Based on the results of an *in silico* analysis, we can conclude that EnSerp1 has structural features that correspond to equivalent structures in other members of the serpin superfamily, especially those involved in the regulation of peptidases of the coagulation cascade and fibrinolysis (antithrombin III, heparin cofactor II and PAI-1). Total absence of experimental data unfortunately prevented us from making a relevant comparison with serpins from other monogenean taxa. The inhibitory effect of rEnSerp1 on the activity of factor Xa and plasmin, together with the presence of EnSerp1 in the excretory-secretory products of the worm, does, however, suggest that EnSerp1 works as an anti-inflammatory as well as an anticoagulant agent involved in parasite-host interactions.

## Supplementary files

Supplementary file 1: List of serpins used for phylogenetic analysis (PDF 269 KB). Supplementary file 2: Alignment of platyhelminth serpin sequences (TIFF 4750 KB). Supplementary materials are available at https://www.parasite-journal.org/10.1051/parasite/2018062/olm

## Ethics statement

The maintenance and care of experimental animals was carried out in accordance with European Directive 2010/63/EU and Czech Acts 246/1992 Coll. and 359/2012 Coll. which regulate biomedical research involving animals. Experiments were performed with the legal consent of the Professional Ethics Committee of the Faculty of Science, Charles University, Prague, and the Branch for Research and Development of the Ministry of Education, Youth and Sports of the Czech Republic. The animal facility, its equipment, animal welfare, and accompanying services including maintenance of experimental animals, were approved by the Branch of Animal Commodities of the Ministry of Agriculture of the Czech Republic (approval no. 13060/2014-MZE-17214).

## Conflict of interest

The authors declare that they have no competing interests.
