# Supplementary material for: Identification and partial characterization of a novel serpin from Eudiplozoon nipponicum (Monogenea, Polyopisthocotylea)
Source: Parasite. 2018 Dec 5;25:61. doi: 10.1051/parasite/2018062 (PMC6280883; doi:10.1051/parasite/2018062)
Supplement: Supplementary file 1 — List of serpins used for phylogenetic analysis (PDF 269 KB). [file parasite-25-61-s1.pdf]

Supplementary file 1: List of serpins used for phylogenetic analysis

Sequence Identifier	Name	Source organism	Class
MF288891.1	EnSerp1	*Eudiplozoon nipponicum*	MONOGENEA
scf7180006950201-processed-gene-0.1		*Gyrodactylus salaris*	
XP_018646699.1	Serpin, putative	*Schistosoma mansoni*	TREMATODA
XP_018646698.1	Serpin, putative		
XP_018646700.1	Serpin, putative		
XP_018646704.1	Serpin, putative		
CCD60071.1	Serpin, putative		
XP_012797534.1	Serpin B4	*Schistosoma haematobium*	
XP_012797533.1	Serpin B8		
AAK57435.1	Serine protease inhibitor serpin	*Schistosoma japonicum*	
AAW25282.1	SJCHGC02866 protein		
GAA35588.2	Leukocyte elastase inhibitor	*Clonorchis sinensis*	
AHZ96593.1	Serine proteinase inhibitor 3		
GAA28469.2	Serpin B		
ADI60059.1	Serpin		
GAA37554.2	Serpin I2		
XP_009168246.1	Hypothetical protein T265_05071	*Opisthorchis viverrini*	
XP_009177032.1	Hypothetical protein T265_11935		
XP_009169304.1	Hypothetical protein T265_05905		
ABV57466.1	Serine protease inhibitor	*Paragonimus westermani*	
EUB63679.1	Serine protease inhibitor	*Echinococcus granulosus*	CESTODA
CDS22753.1	Serine protease inhibitor		
BAU98534.1	Serine protease inhibitor 2b	*Echinococcus multilocularis*	
BAU98533.1	Serine protease inhibitor 2		
CDS35969.1	Serine protease inhibitor		
ATG83400.1	Serpin	*Taenia solium*	
ATG83397.1	Serpin		
JAP62381.1	Serpin I2 partial	*Schistocephalus solidus*	
JAP54469.1	Serpin B13		
CDS32008.2	Estrogen regulated protein EP45	*Hymenolepis microstoma*	
