# Supplementary figures and images for: Identification and partial characterization of a novel serpin from Eudiplozoon nipponicum (Monogenea, Polyopisthocotylea)
Source: Parasite. 2018 Dec 5;25:61. doi: 10.1051/parasite/2018062 (PMC6280883; doi:10.1051/parasite/2018062)

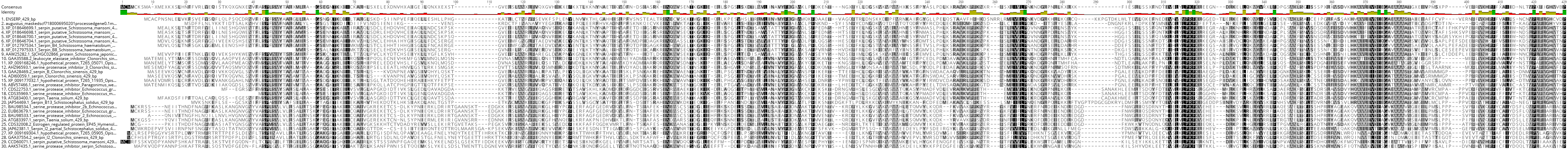

Supplement: Supplementary file 2 — Alignment of platyhelminth serpin sequences (TIFF 4750 KB). [file parasite-25-61-s2.tiff]
